# Evaluation of 1-year follow-up results of macular telangiectasia type 2 cases by optical coherence tomography angiography

**DOI:** 10.3205/oc000118

**Published:** 2019-08-20

**Authors:** Semra Tiryaki Demir, Dilek Güven, Mehmet Egemen Karatas, Ayse Burcu Dirim, Selam Yekta Sendül, Melih Ustaoglu

**Affiliations:** 1 Department of Ophthalmology, Şişli Hamidiye Etfal Training and Research Hospital, İstanbul, Turkey

**Keywords:** vessel densities, superficial capillary plexus, deep capillary plexus

## Abstract

The results of 1-year follow-up with optical coherence tomography angiography (OCTA) of 3 patients with macular telangiectasia type 2 (MacTel 2) were evaluated. The 3X3 mm OCTA imaging was performed in January 2017 and February 2018. The superficial and deep capillary plexus vascular density changes of the whole area, the parafoveal temporal and parafoveal nasal areas were examined. The mean whole, parafoveal temporal, and parafoveal nasal vessel densities at superficial capillary plexus were 51.31, 50.39, 54.57 at baseline and 49.93, 46.79, 51.83 at 1-year follow-up, respectively. The mean whole, parafoveal temporal and parafoveal nasal vessel densities at deep capillary plexus were 59.06, 59.05, 63.39 at baseline and 52.18, 54.68, 57.9 at 1-year follow-up, respectively. In this case series, it was shown quantitatively that vessel densities of MacTel2 patients markedly decreased over time, more pronounced in the deep capillary plexus.

## Introduction

Macular telangiectasia type 2 (MacTel2) is a bilateral retinal disease with characteristic alterations of the macular capillary network and neurosensory atrophy. It primarily affects the juxtafoveal region of the macula [1].

Optical coherence tomography angiography (OCTA) is a non-invasive imaging method for visualization of the retinal capillary network [2]. In recent years, OCTA has been examined as potential imaging modality to facilitate the diagnosis and characterization of MacTel2 [3].

In this case series, we aimed to evaluate the 1-year follow-up results of MacTel2 patients by OCTA.

## Case descriptions

The MacTel2 patients who were followed in our clinic and diagnosed by fundus examination, fluorescein angiography (FA), and optical coherence tomography (OCT) were evaluated. The age and best-corrected visual acuity (VA) according to the Snellen chart of the patients were recorded. The eyes were divided into two groups as proliferative and non-proliferative MacTel2 [4].

The OCTA imaging (Optovue RTVue XR 100 Avanti, Fremont, California, USA) was performed in January 2017 and February 2018. A circular area with a diameter of 3 mm centered on the fovea was examined and vascular densities (VD) of this circular area were calculated by the automated software [5]. The VD (%) of the superficial (SCP) and deep capillary plexus (DCP) were determined. Between baseline and 1-year follow-up results, changes in the VD in the whole, the parafoveal temporal and parafoveal nasal regions were evaluated.

Six eyes of three female patients were included. The mean age was 65.3±11.37. The mean VA was 0.45±0.36 at baseline and remained stable at 1-year follow-up. The VD of SCP and DCP of the cases are shown at baseline and at 1-year follow-up (Table 1 (Tab. 1)). The mean whole, parafoveal temporal, and parafoveal nasal VD at SCP were 51.31, 50.39, 54.57 at baseline and 49.93, 46.79, 51.83 at 1-year follow-up. The mean whole, parafoveal temporal, and parafoveal nasal VD at DCP were 59.06, 59.05, 63.39 at baseline and 52.18, 54.68, 57.9 at 1-year follow-up.

### Case 1 

**Figure 1:**
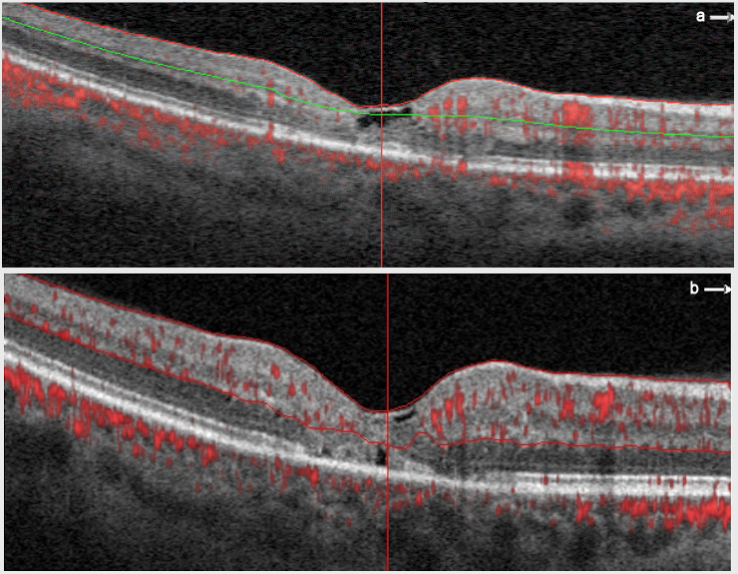
Horizontal B-scan optical coherence tomography angiography (OCTA) image of the right eye of case 1 at baseline (a) and at at 1-year follow-up (b). a. OCTA image shows multiple, telangiectatic, microaneurysmal-like dilated vessels residing within the middle retinal layer and extending to the inner and outer retina in nasal juxtafoveal network. The vascular density of the temporal juxtafoveal network is less than the nasal juxtafoveal network. b. There seems to be a decrease in vessel density.

A 51-year-old female presented with VA of 0.3 in the right eye (OD) and 0.05 in the left eye (OS). The non-proliferative MacTel2 was present in both eyes (OU) and was followed for 2 years. The VA of OU was stable for 1 year and no treatment was performed. The OCTA images of OD and image features are shown at baseline and 1-year follow-up (Figure 1a,b (Fig. 1)).

### Case 2 

**Figure 2:**
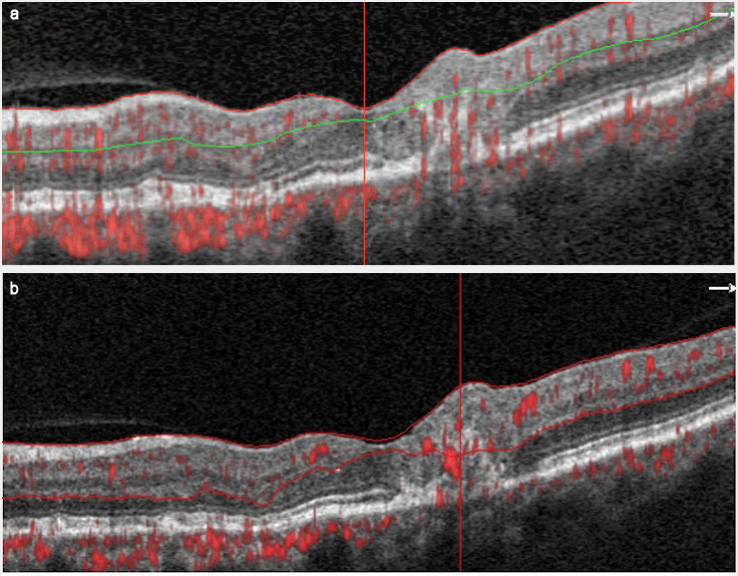
Horizontal B-scan optical coherence tomography angiography (OCTA) image of the left eye of case 2 at baseline (a) and at at 1-year follow-up (b). Both OCTA images show significant alterations in the temporal juxtafoveal network with prominent anastomoses. The presence of abnormal vessels corresponds to an area with retinal vascular anastomoses that extends to the outer retina where the elipsoid zone is disrupted, forming a subretinal neovascularization.

A 78-year-old female presented with VA of 0.8 OD and 1.0 OS. Non-proliferative MacTel2 OD and proliferative MacTel2 OS were followed for 3 years. Intravitreal ranibizumab injection was applied 7 times due to foveal retinal hemorrhage OS. The VA of the patient was stable for 1 year and no additional treatment was given. The OCTA images of OS and image features are shown at baseline and 1-year follow-up (Figure 2a,b (Fig. 2)). 

### Case 3 

**Figure 3:**
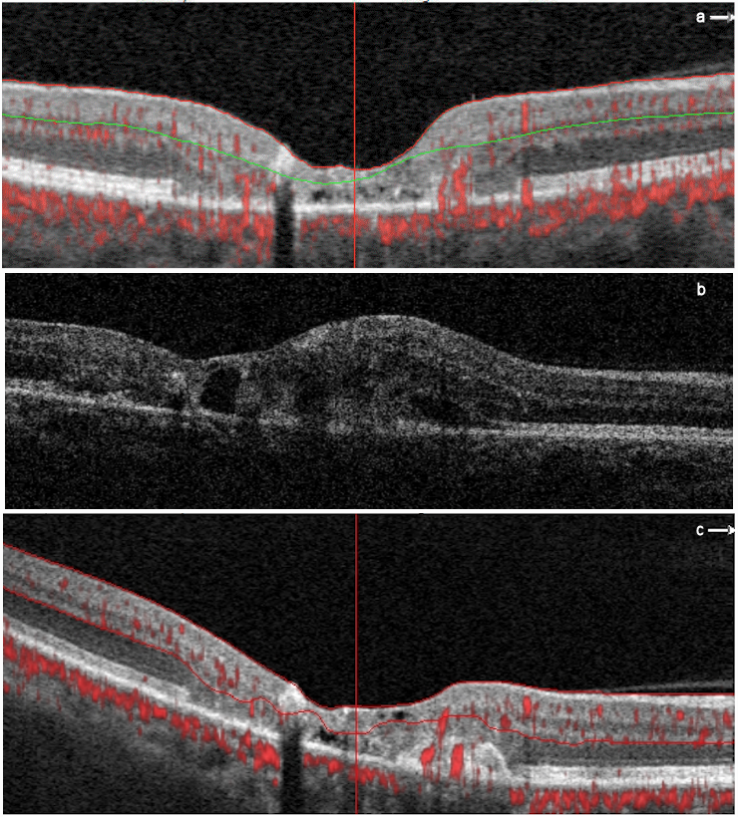
Horizontal B-scan optical coherence tomography angiography (OCTA) and OCT images of the right eye of case 3 at baseline, 6-month and 1-year follow-up. a. Horizontal B-scan OCTA image at baseline. Although no findings were found in OCT, there is a prominent anastomoses in vascular the network between the outer retina and the choriocapillaris layers in the nasal juxtafoveal network in OCTA. b. OCT image at 6-month follow-up. There are intraretinal hyporeflective cavitation, retinal pigment epithelium migration creating intraretinal hyperreflectivity, atrophy of the outer retinal layers, subretinal fluid, and subretinal neovascularization in the fovea. c. Horizontal B-scan OCTA image at 1-year follow-up.

A 56-year-old female presented with VA of 0.3 OD and 0.4 OS. The non-proliferative MacTel2 was present OU, was followed for 7 years and no treatment was performed. Six months after the first OCTA, VA of OD was 0.15. Fundus examination revealed foveal retinal hemorrhage OD. The OCT image of OD and image features are shown at 6 months (Figure 3b (Fig. 3)). Monthly intravitreal bevacizumab injections were applied to OD 5 times due to subretinal neovascularization (SRN). After 6 months, VA increased to 0.3 and retinal hemorrhage completely disappeared. The VA of OS was stable within 1 year. The OCTA images of OD and image features are shown at baseline and 1-year follow-up (Figure 3a,c (Fig. 3)).

## Discussion

The MacTel2 is a neurodegenerative and vascular disease limited to the macular area. Dysfunction and loss of Muller cells play an important role [6]. Loss of regulation of these cells may lead to photoreceptor death, dysregulation of angiogenic factors, vascular ectasia, and subsequent SRN [7].

OCTA has been recently used to analyze the macular capillary network in this disease [8]. OCTA has shown an increase in the intervascular spaces with progressive capillary rarefaction and abnormal capillary anastomosis and a vascular invasion in the normally avascular outer retina [9], [10]. Early changes in the retinal microvasculature begin temporal to the fovea in the DCP and then extend into the SCP [7], [8], [9], [10], [11]. OCTA imaging not only helps to facilitate the early diagnosis of MacTel2 but also provides a better understanding of disease progression and treatment efficacy [12].

This case series presents OCTA imaging features of non-proliferative and proliferative MacTel2 at 1-year follow-up. Proliferative and non-proliferative MacTel2 eyes showed marked reduction of VD of SCP and DCP at 1-year follow-up. The decrease in VD of DCP was greater than of SCP. For this reason, we think that ischemia may play a very important role in the pathogenesis and progression of MacTel2. We think that retinal atrophy and SRN may develop due to ischemia. 

We did not use anti-vascular endothelial growth factor (VEGF) therapy because there was no decrease in VA of the patients with proliferative MacTel2 within 1 year, despite the detection of SRN in OCTA. One eye progressed to proliferative MacTel2 within 1 year. Although no findings were found in OCT, there was a prominent anastomosis in the vascular network between the outer retina and the choriocapillaris layers in OCTA at baseline. The VA decreased after 6 months. Despite anti-VEGF treatment, there is still SRN with high vascular density in OCTA. Therefore, if there is no decrease in VA, we think that anti-VEGF therapy is not necessary, even if the SRN is present in the OCTA, because it can increase retinal atrophy [13].

## Conclusions

Based on the presented cases, the VD of MacTel2 patients might be decreased over time, more pronounced in the DCP. The VD of SCP and DCP may be an important parameter in long-term follow-up of MacTel2 patients. OCTA imaging provides a better understanding of disease progression. However, the presence of SRN with high VD in OCTA does not indicate that anti-VEGF therapy is required. Future studies with OCTA will hopefully illuminate additional features of MacTel2 and provide help in assessing treatment outcomes.

## Notes

### Competing interests

The authors declare that they have no competing interests.

## Figures and Tables

**Table 1:**
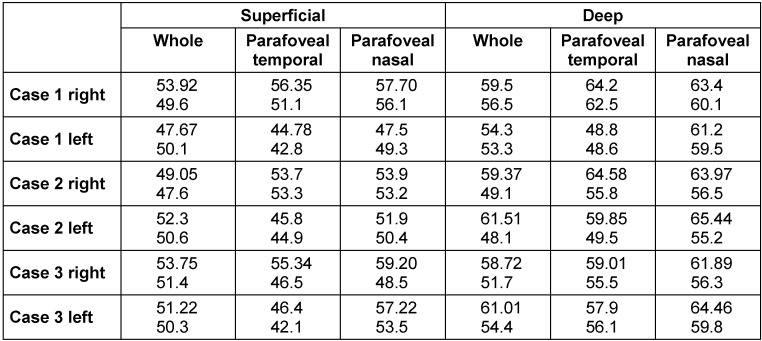
Vessel densities (%) of superficial and deep capillary plexus of the cases in en face OCTA are shown at baseline (upper) and 1-year follow-up (bottom).
